# MCPIP1 contributes to the toxicity of proteasome inhibitor MG-132 in HeLa cells by the inhibition of NF-κB

**DOI:** 10.1007/s11010-014-2134-z

**Published:** 2014-07-04

**Authors:** Lukasz Skalniak, Monika Dziendziel, Jolanta Jura

**Affiliations:** Department of General Biochemistry, Faculty of Biochemistry, Biophysics and Biotechnology, Jagiellonian University, ul. Gronostajowa 7, 30-387 Kraków, Poland

**Keywords:** MCPIP1, ZC3H12A, Regnase, MG-132, NF-kappaB, MG132

## Abstract

Recently, we have shown that the treatment of cells with proteasome inhibitor MG-132 results in the induction of expression of monocyte chemotactic protein-1 induced protein 1 (MCPIP1). MCPIP1 is a ribonuclease, responsible for the degradation of transcripts encoding certain pro-inflammatory cytokines. The protein is also known as an inhibitor of NF-κB transcription factor. Thanks to its molecular properties, MCPIP1 is considered as a regulator of inflammation, differentiation, and survival. Using siRNA technology, we show here that MCPIP1 expression contributes to the toxic properties of MG-132 in HeLa cells. The inhibition of proteasome by MG-132 and epoxomicin markedly increased MCPIP1 expression. While MG-132 induces HeLa cell death, down-regulation of MCPIP1 expression by siRNA partially protects HeLa cells from MG-132 toxicity and restores Nuclear factor-κB (NF-κB) activity, inhibited by MG-132 treatment. Inversely, overexpression of MCPIP1 decreased constitutive activity of NF-κB and limited the survival of HeLa cells, as we have shown in the previous study. Interestingly, although MG-132 decreased the expression of IκBα and increased p65 phosphorylation, the inhibition of constitutive NF-κB activity was observed in MG-132-treated cells. Since the elevated constitutive activity of NF-κB is one of the mechanisms providing increased survival of cancer cells, including HeLa cells, we propose that death-promoting properties of MCPIP1 in MG-132-treated HeLa cells may, at least partially, derive from the negative effect on the constitutive NF-κB activity.

## Introduction

 Monocyte chemotactic protein-1 induced protein 1 (MCPIP1) is a multifunctional protein involved in the regulation of such processes as macrophage activation [1, 2], cell differentiation [3, 4], and survival [5–7] (reviewed in [8]). The protein was shown to negatively regulate inflammation-related processes by the inhibition of Nuclear factor-κB (NF-κB) transcription factor [1, 9] and endoribonucleolytic degradation of transcripts encoding pro-inflammatory cytokines, i.e., interleukin (IL)-6, IL-1β, IL12b, and IL-2 [10–12]. Recently, we have shown that the expression of MCPIP1 is markedly increased in HeLa and HepG2 cells upon the treatment with proteasome inhibitor MG-132 [13]. We have also shown that MCPIP1 overexpression decreases the survival of HeLa cells [13]. Our initial data suggested that MCPIP1 expression may partially be involved in toxic properties of MG-132 proteasome inhibitor.

The inhibition of proteasome became an extensively studied strategy in cancer treatment due to the importance of ubiquitin–proteasome system in the promotion of cell cycle progression and activation of NF-κB signaling pathway as well as removal of tumor suppressor protein p53 [14]. Within past years, numerous studies showed the astonishing potency of proteasome inhibitors to induce cancer cell death. Up to date, thousands of proteasome inhibitors have been developed (reviewed in [15]), among which several entered clinical trials, and two, namely bortezomib (PS-341/Velcade) [16] and carfilzomib [17], have been approved by *US Food and Drug Administration* for the treatment of multiple myeloma patients [18–20]. The mechanism of proteasome inhibition-induced toxicity relies on several events, among which the induction of endoplasmic reticulum stress and unfolded protein response play a major role, with accompanying deleterious functions of reactive oxygen species production, NF-κB inhibition and inhibition of the degradation of cell cycle regulators and pro-apoptotic factors [21–23].

The exact effect of proteasome inhibition on NF-κB activity requires deeper verification since up-to-date reports seem to be contradictory. Many studies proved for the inhibitory effect of such compounds as bortezomib or MG-132 on TNFα and IL-1β-induced NF-κB [24–26] or constitutive NF-κB activity [27, 28]. Contrary to these findings, several reports pointed that both bortezomib and MG-132 alone may lead to the degradation of IκBα protein [29–31] and activation of NF-κB [29, 32]. In the present study, we verify the impact of proteasome inhibitor MG-132 on IκBα expression and the activity of NF-κB transcription factor. We also correlate our observations with the induction of MCPIP1 expression upon MG-132 treatment and with the deleterious character of this protein toward tested HeLa cell line.

## Results

### Proteasome inhibitor epoxomicin increases the expression of MCPIP1

**Fig. 1:**
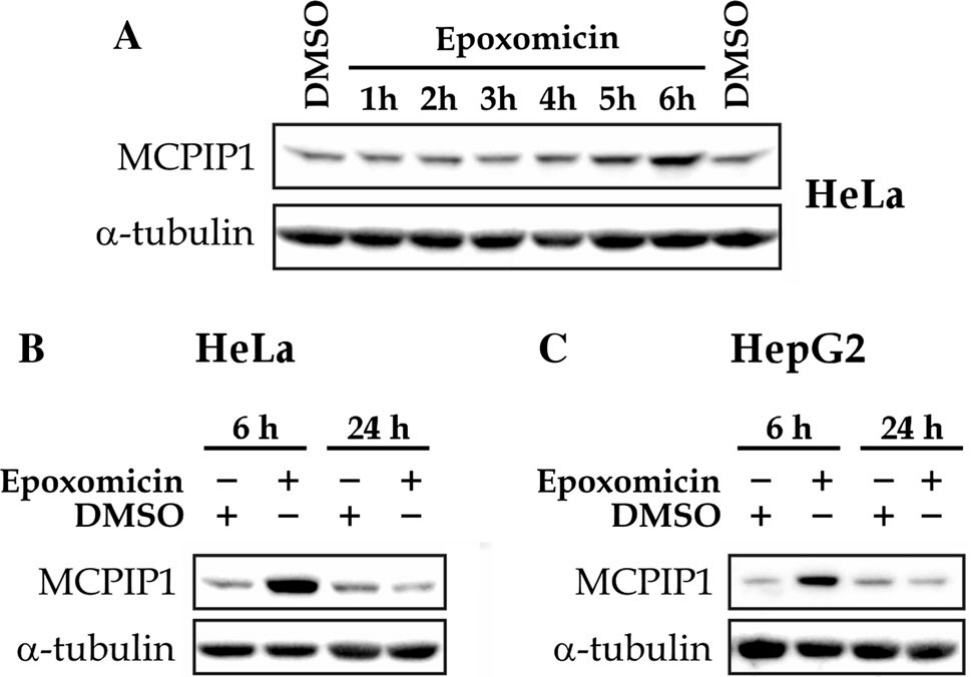
Proteasome inhibitor, epoxomicin, increases the expression of MCPIP1.** a**–**c** HepG2 or HeLa cells (as indicated) were treated with 100 nM epoxomicin or DMSO for the indicated time periods. Protein extracts were subjected to western blotting with antibodies specific for MCPIP1 and α-tubulin. Blots are representative from three independent experiments

We have shown recently that the inhibition of proteasome with MG-132 results in transcription-dependent increase of MCPIP1 protein [13]. Besides the inhibition of proteasome, at the higher concentrations MG-132 (ZLLLal) inhibits calpains and cathepsins [15, 33]. Although the dose used in our study (1 µM) was below IC_50_ of calpain inhibition, we verified the observed effect of MG-132 on MCPIP1 expression level using epoxomicin—the most specific and potent proteasome inhibitor known [15]. Epoxomicin increased the expression of MCPIP1 protein in HeLa cells at the time points 5 and 6 h of the treatment (Fig. 1a, b). Similarly, in HepG2 cells the increase of MCPIP1 expression was observed after 6 h of epoxomicin treatment (Fig. 1c). Interestingly, unlike in the case of MG-132 [13], the level of MCPIP1 expression dropped down to the basal level after 24 h since the administration of epoxomicin in both tested cell lines (Fig. 1b, c).

### Proteasome inhibition by MG-132 results in the phosphorylation-dependent degradation of IκBα

**Fig. 2:**
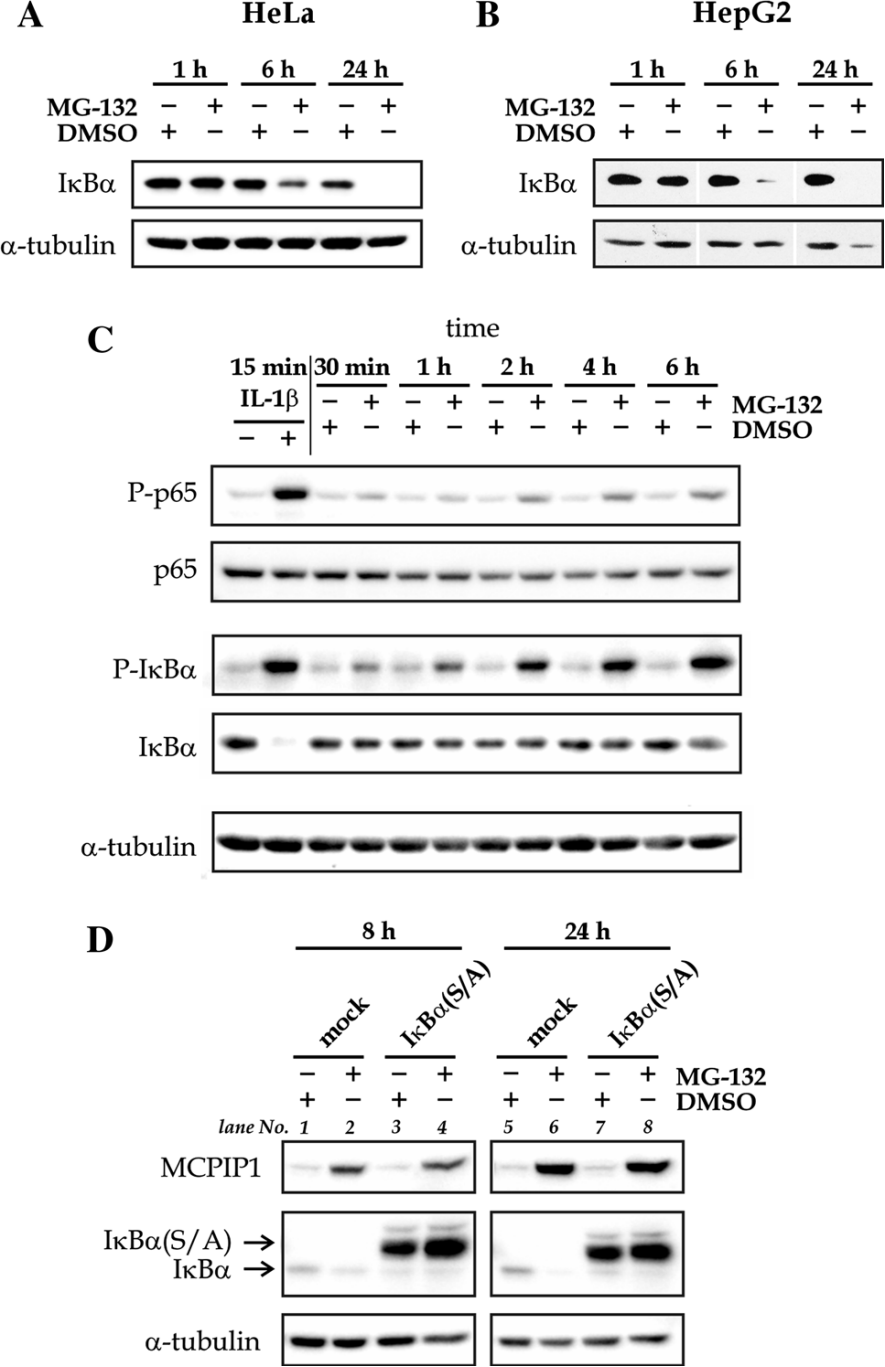
Proteasome inhibitor MG-132 leads to phosphorylation-dependent removal of IκBα.** a**,** b** HepG2 or HeLa cells (as indicated) were treated with 1 µM of MG-132 or DMSO for the indicated time periods and subjected to western blotting against IκBα and α-tubulin. **c** HeLa cells were treated with 10 ng/ml of IL-1β, 1 µM of MG-132 or DMSO for the indicated time periods and subjected to western blotting against P-p65, p65, P-IκBα, IκBα, and α-tubulin. **d** Two HepG2 stable cell lines: IκBα(S/A), overexpressing dominant negative mutant of IκBα protein deprived of phosphorylation sites Ser32 and Ser36, and control mock cell lines were treated with MG-132 or DMSO for 8 or 24 h. Protein lysates were collected and subjected to western blotting with the indicated antibodies. The blots presented here are representative of three experiments

It has been reported that prolonged inhibition of proteasome with bortezomib or MG-132 results in caspase-8 and/or calpain-dependent degradation of IκBα [30, 31]. To verify the impact of MG-132 on IκBα expression, HepG2 and HeLa cells were treated with 1 µM MG-132 for 1, 6, or 24 h. The treatment of the cells for 1 h did not change the expression level of IκBα (Fig. 2a, b). In contrast, exposition of cells to MG-132 for 6 h resulted in a decrease of IκBα quantity in both cell lines tested, and the treatment of cells for 24 h resulted in a total disappearance of IκBα (Fig. 2a, b).

Consecutive phosphorylation, ubiquitination, and proteasomal degradation of IκBα are important events for the activation of the canonical NF-κB pathway. To verify if MG-132 treatment increases IκBα phosphorylation prior to the protein disappearance, HeLa cells were treated with 1 µM MG-132 for 30 min or 1, 2, 4, or 6 h, and western blot detection of phospho-IκBα was performed (Fig. 2c). The stimulation with 10 ng/ml of IL-1 β for 15 min, serving as a positive control, resulted in phosphorylation of IκBα and its almost complete removal (Fig. 2c). The treatment of HeLa cells with MG-132 strongly and time-dependently increased the phosphorylation of IκBα (Fig. 2c). Additionally, as a hallmark of NF-κB activation, we verified the phosphorylation of p65 subunit of NF-κB at the position Ser536. Both IL-1β and MG-132 treatment resulted in the phosphorylation of p65 (Fig. 2c). The phosphorylation of p65 in MG-132-treated cells developed with time but at the longest time tested (6 h) was at least four times weaker than phosphorylation observed in IL-1β-treated cells (Fig. 2c).

Previously, it has been proposed that the mutant variant of IκBα deprived of two phosphorylation sites at the positions Ser32 and Ser36, which is resistant to proteasome-mediated degradation, is also resistant to MG-132-triggered degradation [31]. To test this, we took advantage of two cell lines stably transduced with an empty virus (HepG2-mock cell line) or a virus encoding IκBα(S/A)—a mutant of IκBα protein bearing two mutations at the mentioned serine residues (HepG2- IκBα(S/A) cell line) [34]. Overexpression of the IκBα(S/A) mutant resulted in the decrease of endogenous IκBα expression when compared to untreated mock cell line (Fig. 2d, lanes 3 and 4 compared to 1 and lanes 7 and 8 compared to 5).The treatment of HepG2-mock cells with MG-132 decreased the level of endogenous IκBα expression, both after 8 and 24 h of treatment (Fig. 2d, lanes 2 and 6 compared to 1 and 5, respectively). In HepG2-IκBα(S/A) cell line, a strong IκBα-specific signal was observed just above the signal corresponding to endogenous IκBα, reflecting the expression of the transgene (IκBα(S/A) protein). The expression of IκBα(S/A) was not decreased following MG-132 treatment, which suggests that the IκBα mutant is indeed resistant to the degradation observed after prolonged proteasome inhibition (Fig. 2d, lanes 4 and 8 compared to 3 and 7, respectively).

Upon the treatment of HepG2-mock cells with MG-132, a strong increase of MCPIP1 expression was observed at both time points tested: 8 and 24 h (Fig. 2d). Similarly, in HepG2-IκBα(S/A) cells MCPIP1 expression increase, comparable to mock cell line, was observed (Fig. 2d).

### The inhibition of proteasome by MG-132 decreases constitutive activity of NF-κB

The removal of IκBα and phosphorylation of the p65 subunit following MG-132 treatment suggest the possibility of NF-κB activation. In order to verify the activity of NF-κB following MG-132 treatment, a secreted alkaline phosphatase (SEAP)-based NF-κB promoter activity test was performed on cells transfected with a vector containing a SEAP-coding gene under the control of four NF-κB-binding sites (plasmid pNFκB-SEAP). The cells were treated with DMSO or MG-132 for 2, 4, 6, 8, 16, or 24 h, and the accumulation of SEAP in media was measured using luminescence-based method.

**Fig. 3:**
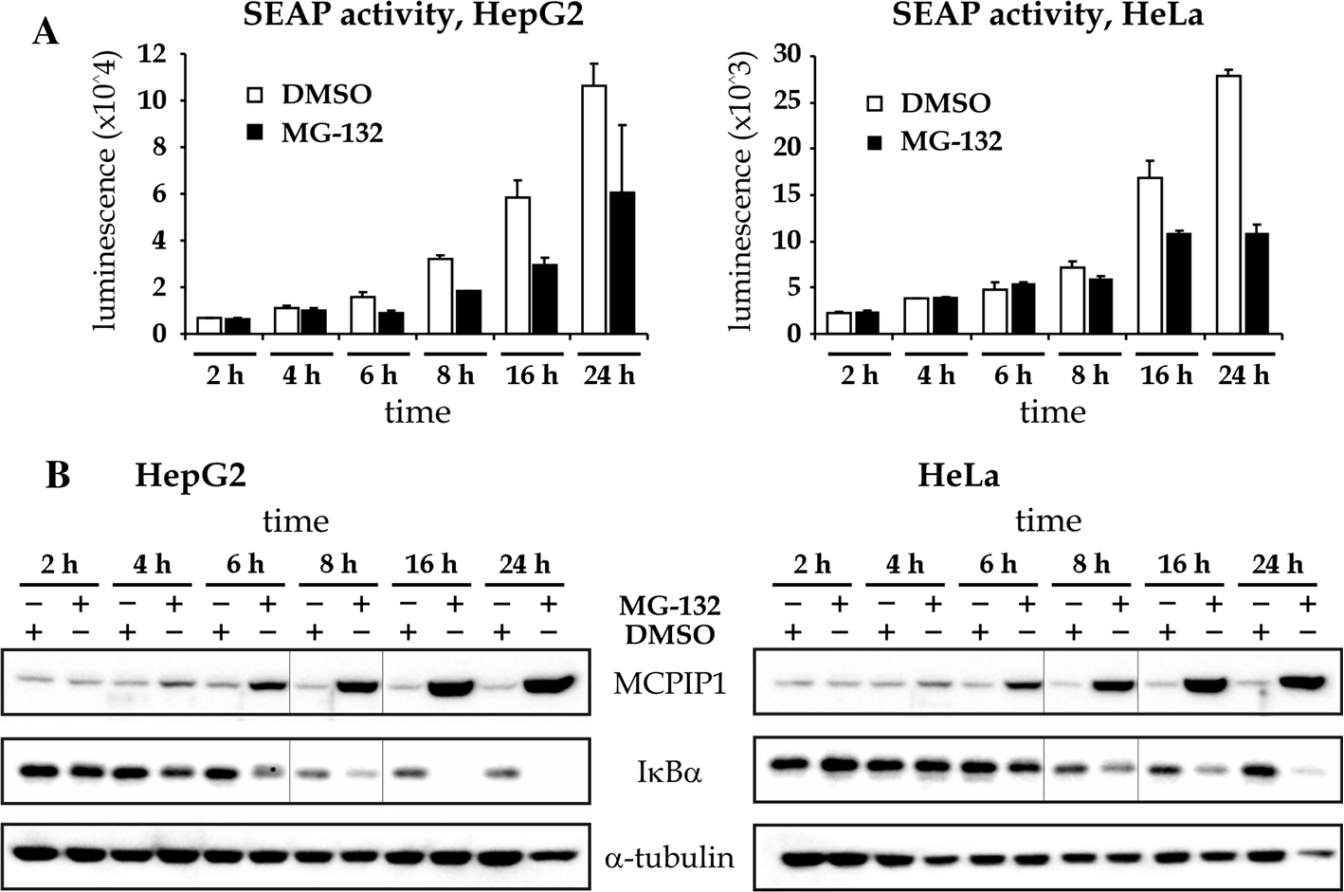
MG-132 inhibits NF-κB-dependent expression. **a** HepG2 or HeLa cells were transfected with the plasmid containing SEAP-encoding gene under the control of four κB sites (pNFκB-SEAP vector). After 48 h, the cells were treated with 1 µM of MG-132 for the indicated time periods. Media were collected for the measurement of accumulated SEAP activity and the cells were lysed and subjected to western blotting with antibodies specific for MCPIP1, IκBα, and α-tubulin (**b**). Graphs show fold change of SEAP activity versus DMSO-treated control at the time point 2 h and are mean ± SE from three experiments. Blots are representative from two experiments

The activity of SEAP in culture media from DMSO-treated cells increased with time due to a constitutive NF-κB activity (Fig. 3a, white bars). Interestingly, the treatment of cells with MG-132 resulted in 20–60 % lower accumulation of SEAP starting from the 6th hour of the treatment in case of HepG2 cells and the 8th hour of treatment in case of HeLa cells (Fig. 3a, black bars versus white bars). This suggests the inhibition of NF-κB by prolonged MG-132 treatment rather than its activation. To verify the expression of MCPIP1 and IκBα in cells transfected with SEAP-coding plasmid, a western blot was performed. As expected, the treatment resulted in a time-dependent increase of MCPIP1 protein and time-dependent decrease of IκBα, in accordance with the results presented in previous sections (Fig. 3b).

### MCPIP1 decreases both constitutive and IL-1β-induced transcriptional activity of NF-κB

**Fig. 4:**
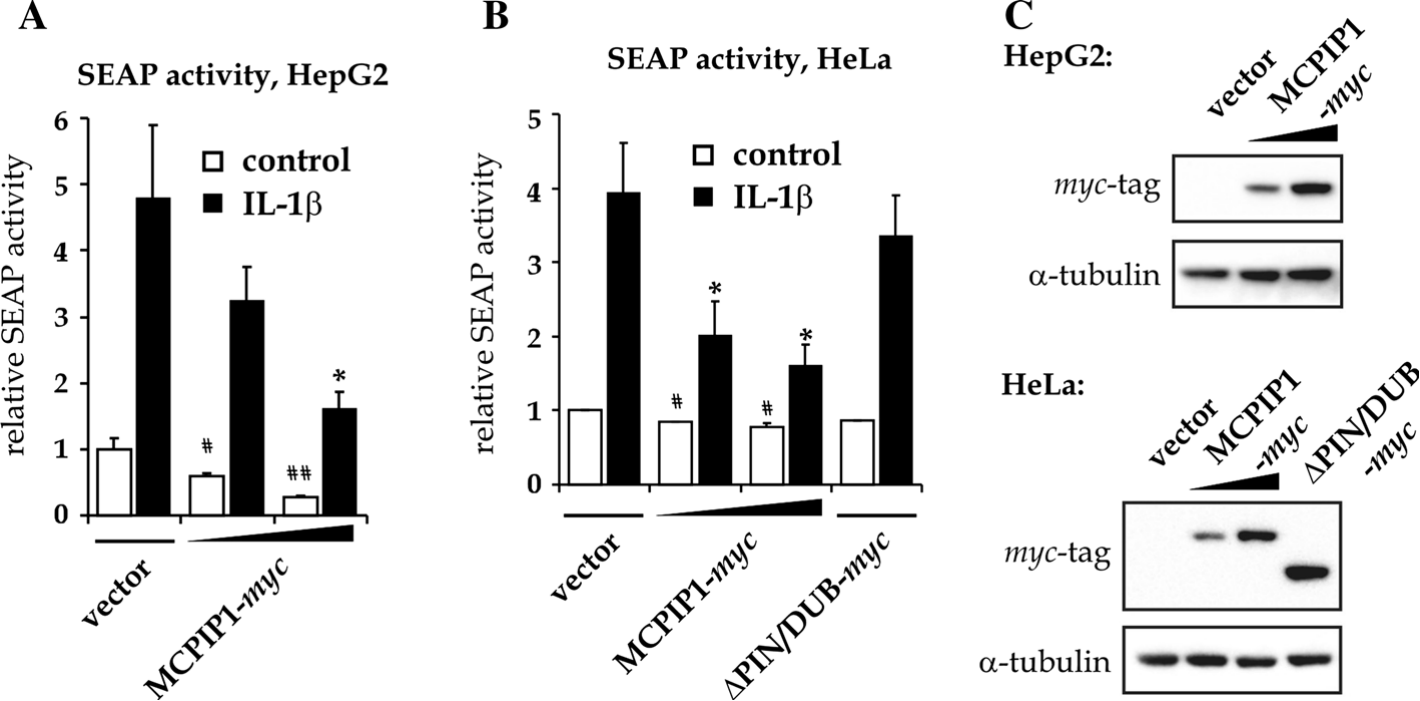
MCPIP1 inhibits NF-κB-dependent expression of reporter gene, both at the constitutive and IL-1β-induced NF-κB activity level. HepG2 cells **a** or HeLa cells **b** were transfected with pNFκB-SEAP vector together with plasmids encoding MCPIP1-*myc* or ΔPIN/DUB-*myc* protein or an empty vector. Two days following transfection the cells were stimulated with 10 ng/ml of IL-1β for 24 h. Media were collected and luminescence-based SEAP activity assay was performed. The expression of MCPIP1 and ΔPIN/DUB-*myc* in tested cells was verified by western blot (**c**). Graphs show means ± SE from three experiments, presented as fold change versus empty vector-transfected control. For the statistics *t* test was performed: *P < 0.05 versus vector-transfected IL-1β-treated cells;^ #^ *P* < 0.05,^ ##^ *P* < 0.01 versus vector-transfected DMSO-treated control cells

Previously, we and others have reported that MCPIP1 inhibits NF-κB transcription factor activity [1, 9]. The NF-κB-inhibitory properties of MCPIP1 have been assigned to the enzymatic domain named PIN/DUB [35]. To additionally verify the effect of MCPIP1 on NF-κB-driven transcription and to test the requirement of PIN/DUB domain in this process, a SEAP activity test was performed. For this, HepG2 and HeLa cells were co-transfected with the pNFκB-SEAP vector and two amounts of plasmid encoding MCPIP1-*myc* protein, the protein variant deprived of PIN/DUB domain (ΔPIN/DUB-*myc*) or an empty vector. To minimize the toxic properties of MCPIP1 in transfected cells, the amount of plasmid encoding MCPIP1 proteins was limited to 15 and 30 % of total DNA used in transfection. The mixture of SEAP-coding plasmid and empty plasmid constituted the remaining 85 or 70 %. The cells were stimulated with IL-1β for 24 h and SEAP accumulation in the medium was measured using luminescence-based assay. The expression of MCPIP1-*myc* and ΔPIN/DUB-*myc* proteins in transfected cells was verified by western blot (Fig. 4c).

The stimulation of HepG2 and HeLa cells transfected with an empty vector with IL-1β resulted in a 5- and 4-fold increase of SEAP activity in the medium, respectively (Fig. 4a, b). This SEAP activity increase was inhibited very strongly and dose-dependently by the expression of MCPIP1-*myc* protein (Fig. 4a, b). In contrast, the expression of ΔPIN/DUB-*myc* protein in HeLa cells failed to inhibit the effect of IL-1β on NF-κB-dependent SEAP accumulation (Fig. 4b).

Notably, besides IL-1β-stimulated NF-κB-dependent expression, the presence of MCPIP1-*myc* protein significantly and dose-dependently decreased also the basal level of SEAP activity in both cell lines tested, suggesting the involvement of MCPIP1 in the inhibition of constitutive NF-κB activity (Fig. 4a, b).

### MCPIP1 silencing increases NF-κB activity and partially protects HeLa cells from MG-132 toxicity

**Fig. 5:**
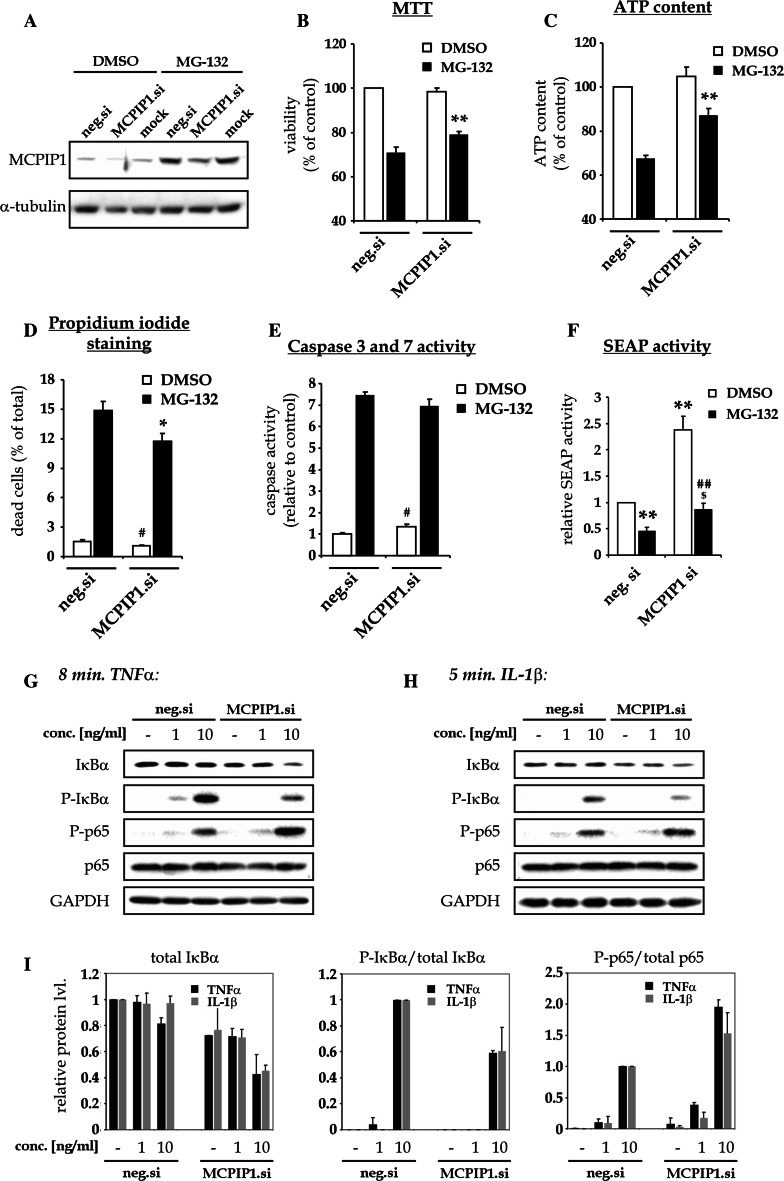
MCPIP1 silencing partially protects HeLa cells against MG-132 toxicity and inhibition of constitutive NF-κB activity**. a**–**e** For the study of the involvement of MCPIP1 in MG-132 toxicity HeLa cells were transfected with MCPIP1-silencing siRNA (MCPIP1.si) or negative control siRNA (neg.si). Cells were cultured for 3 days on culture dish and seeded for the experiments. Next day, the cells were subjected to downstream procedures. **a** The silencing of MCPIP1 was verified by western blot in the cells treated with DMSO or MG-132 for 6 h. **b**–**e** siRNA-transfected cells were treated for additional 24 h with DMSO or 1 µM MG-132, followed by MTT test (**b**) and ATP content analysis (**c**). **d** Following the treatment the cells were double-stained with Hoechst and propidium iodide. The graph shows the percentage of dead cells within total population analyzed. **e** Following the treatment caspase 3/7 activity was measured using luminescence-based assay. Graphs **b**–**e** represent mean ± SE from three experiments, each performed at least in triplicates. For the statistics, *t*-test was performed: *P < 0.05 versus neg.si-transfected MG-132-treated cells, **P < 0.01 versus neg.si1-transfected MG-132-treated cells,^ #^ *P* < 0.05 versus neg.si1-transfected control DMSO-treated cells, *n* = 3. **f** HeLa cells were transfected with negative siRNA (neg.si) or siRNA for MCPIP1 (MCPIP1.si). Next day the cells were transfected with pNFκB-SEAP reporter vector and cultured for 24 h. Then, the cells were treated with DMSO or 1 µM MG-132 for additional 24 h before SEAP activity measurement. Graph shows mean ± SE from three experiments, each performed in triplicates. For the statistics *t*-test was performed: **P < 0.01 versus neg.si-transfected DMSO-treated cells,^ ##^ *P* < 0.01 versus MCPIP1.si-transfected DMSO-treated cells,^ $^ *P* < 0.05 versus neg.si1-transfected MG-132-treated cells, *n* = 3. **g**–**i** MCPIP1 silencing improves the induction of early stages of cytokine-induced NF-κB activation. HeLa cells were treated with siRNA and cultured for 3 days as described in figures **a**–**e** caption. The cells were seeded and, after 24 h, stimulated with the indicated concentrations of cytokines for 8 min (TNFα) or 5 min (IL-1β). Whole cell lysates were subjected to western bloting with indicated antibodies. The blots **g**, **h** are representative of two experiments. **i** semi-quantification of blots **g**, **h** normalized to untreated control (for total IκBα) or control cells treated with 10 ng/ml of cytokine (P-IκBα/total IκBα and P-p65/total p65). Graphs show mean ± SE from two experiments

As we have shown previously increased expression of MCPIP1 decreases the survival of HeLa cells [13]. In order to verify, the involvement of the elevated MCPIP1 in the toxicity of MG-132 toward HeLa cells siRNA technology was applied. For this, HeLa cells cultured on Ø 60 mm culture dishes were transfected with negative siRNA (depicted as neg.si) or MCPIP1 siRNA (depicted as MCPIP1.si) and cultured for 72 h. Then, the cells were seeded on culture plates for the experiments. Next day, cells were treated with 1 µM of MG-132 and survival and toxicity were measured after 24 h using MTT test, ATP content, propidium iodide/Hoechst double staining, and caspase 3/7 activity (Fig. 5). Parallely, the silencing of MCPIP1 expression was verified in siRNA-transfected and cultured cells by western blotting, after 6 h of treatment with DMSO or 1 µM MG-132 (Fig. 5a).

The treatment of neg.si1-transfected control HeLa cells with 1 µM MG-132 decreased the viability of cells by around 30 %, when analyzed by MTT or ATP content test (Fig. 5b, c). MG-132 treatment resulted in a 10-fold increase of the number of dead propidium iodide-positive cells, from 1.5 to 15 %, and resulted in a 7.5-fold increase of the activity of caspase 3/7 (Fig. 5d, e). siRNA-mediated down-regulation of MCPIP1 expression in HeLa cells did not significantly change the basal metabolism nor viability, as revealed by MTT test and ATP content measurement (Fig. 5b, c, white bars, *P* = 0.26, *n* = 4 and *P* = 0.15, *n* = 3 for MTT and ATP content respectively). In contrast, propidium iodide staining revealed a faint but significant decrease of dead cells after MCPIP1 silencing (Fig. 5d, white bars), while a weak but significant increase of caspase 3/7 activity was observed in MCPIP1.si- versus neg.si-transfected cells (Fig. 5e, white bars).

More importantly, knock-down of MCPIP1 by siRNA resulted in a significant protection of HeLa cells against MG-132 toxicity. In MTT test, the viability of analyzed cells was increased by 8 % and in ATP content test by 20 % (Fig. 5b, c, black bars). MCPIP1-specific siRNA decreased also the number of propidium iodide-positive cells from 14.9 to 11.7 % after MG-132 treatment (Fig. 5d, black bars). No significant change in caspase 3/7 activation was observed in the cells treated with MCPIP1.si in comparison to neg.si-transfected cells (Fig. 5e, black bars, *P* = 0.20, *n* = 3).

MCPIP1 is known to inhibit NF-κB activation. To verify the engagement of elevated MCPIP1 in NF-κB inactivation by MG-132, HeLa cells were transfected with MCPIP1.si or neg.si siRNAs together with pNFκB-SEAP reporter vector. Next day, the cells were treated with MG-132 inhibitor, and SEAP activity test was performed.

Silencing of MCPIP1 expression by siRNA resulted in a strong and significant increase of SEAP accumulation in both DMSO- and MG-132-treated cells (Fig. 5f, white bars and black bars). The treatment with MG-132 of both neg.si1-transfected and MCPIP1.si-transfected cells decreased the accumulation of SEAP in the medium by 54 and 64 %, respectively (Fig. 5f). Importantly, in MCPIP1-silenced and MG-132-treated cells, the measured activity of SEAP was comparable to its activity in DMSO-treated neg.si-transfected cells, suggesting the restoration of disturbed NF-κB by MCPIP1 down-regulation (Fig. 5f).

To confirm the NF-κB inhibitory properties of MCPIP1 in HeLa cells, we investigated the early stages of the activation of this transcription factor, i.e., IκBα degradation and phosphorylation of IκBα and p65, in MCPIP1-silenced HeLa cells stimulated with TNFα and IL-1β (Fig. 5g–h). Short times of cell stimulation were chosen to avoid: (a) total degradation of IκBα in neg.si-transfected, cytokine-treated cells, and (b) degradation of MCPIP1, which has been recently shown by others in HeLa cells treated with IL-1β [36].

The stimulation of HeLa cells with TNFα and IL-1β resulted in the marked increase of IκBα phosphorylation in both neg.si and MCPIP1.si-transfected cells (Fig. 5g–h). Notably, the degradation of IκBα was not observed in the control cells, suggesting that the time point between IKK activation and IκBα degradation was caught. Importantly, at that time point in MCPIP1.si-transfected cells treated with 10 ng/ml of TNFα or IL-1β already c.a. 40 % of IκBα was degraded. This suggests, that the deprivation of MCPIP1 expression facilitates the activation of NF-κB in HeLa cells following the treatment with each of the two stimulants used.

The decreased amount of P-IκBα in MCPIP1.si-treated cells was observed (Fig. 5g–i). This may be the result of faster removal of phosphorylated IκBα in MCPIP1-deprived cells. Importantly, the phosphorylation of p65 was stronger in MCPIP1-deprived cells, suggesting really stronger NF-κB activation in comparison to neg.si-transfected cells.

Noteworthy, the expression of basal IκBα was lower in MCPIP1.si-transfected cells than in control cells. This may be the symptom of the formation of new regulatory balance in basal NF-κB activity, introduced by the withdrawal of constant inhibition of NF-κB by MCPIP1 in MCPIP1-silenced cells. However, considering the complexity of feedback loops regulating NF-κB, the accurate cause-effect interpretation of this phenomenon is not possible basing on the presented data.

## Discussion

Increased constitutive activity of NF-κB transcription factor is found in a large number [37] if not most tumor cells [38], providing resistance to apoptosis and promoting tumor growth. Initially, it has been proposed that the inhibition of constitutive NF-κB activity is responsible for the observed toxic effects of proteasome inhibition against cancer cells. Nowadays, due to the contradictory reports concerning the impact of proteasome inhibitors on NF-κB activity, this aspect of proteasome inhibition requires profound re-examination.

In our study, the treatment of both HepG2 and HeLa cells with MG-132 led to a disappearance of IκBα protein. This IκBα removal was accompanied by its strong phosphorylation. This phosphorylation seems to be important for the observed degradation of IκBα, since dominant negative variant of this protein, bearing two serine-to-alanine substitution at the phosphorylated positions 32 and 36, was resistant to MG-132-induced degradation. Similar observation was reported before by another group [31]. Thus, upon proteasome inhibition, IκBα undergoes phosphorylation-dependent degradation, but in an impaired fashion, due to the inactivation of ubiquitin–proteasome system. The slow degradation of IκBα in such instance may be executed by caspase 8 [30], calpains [31], remaining proteasome activity or by another, yet undetermined protease.

Besides the phosphorylation of IκBα, MG-132 treatment resulted in the phosphorylation of p65 subunit of NF-κB. Unexpectedly, while both IκBα degradation and p65 phosphorylation are the hallmarks of NF-κB activation, we observed a decrease of NF-κB-driven reporter gene expression in both cell lines treated with MG-132. This suggests that MG-132 does not activate but inhibits constitutive NF-κB activity. This observation seems to be in accordance with a recent report, showing that although another proteasome inhibitor, carfilzomib, induced the disappearance of IκBα, it failed to activate the expression of some tested classical target genes of NF-κB [39]. Moreover, the authors reported no increase in the nuclear localization of NF-κB subunits p50, p65, or p52 [39]. The results presented by these authors, together with our results presented here, indicate that proteasome inhibition leads to IκBα removal and aberrant activation of NF-κB, which does not simply translate into transcriptional activity of this transcription factor.

While it is a surprising but general rule that prolonged inhibition of proteasome leads to the degradation of IκBα, the resulting effect on NF-κB activity seems to be both time- and cell type-dependent. In our study on HeLa and HepG2 cells, decrease of NF-κB activity was observed. A study by Hideshima and co-workers shows that the inhibition of basal NF-κB following bortezomib treatment is observed in cell lines presenting high or moderate constitutive activity of NF-κB (MM.1S and U226), while in the cells with low constitutive NF-κB bortezomib induced the activation of this transcription factor [40]. Importantly, of these three cell lines, MM.1S was shown to be the most susceptible to bortezomib toxicity [41], which may reflect the requirement for NF-κB activity for the cell survival. Similarly, in pancreatic cancer cell line BxPC-3 and squamous cell carcinoma cell line PAM-LY2, which present high level of constitutive NF-κB activity, NF-κB was inhibited by the treatment with MG-132 and bortezomib, respectively [27, 28]. In contrast, the treatment of MIA PaCa 2 cell line (low constitutive NF-κB activity) with MG-132 does not change the observed NF-κB status [28]. Therefore, we postulate that proteasome inhibition leads to the inhibition of constitutive NF-κB in cell lines in which increased constitutive activity of this transcription factor is observed.

We have shown before that the inhibition of proteasome with MG-132 leads to a potent induction of the expression of MCPIP1 in two cell lines analyzed, i.e., HeLa and HepG2 [13]. Here, we support this finding by the use of epoxomicin, the most specific proteasome inhibitor known. MCPIP1 possesses toxic properties as shown in studies on several cell lines [3, 5, 42], including HeLa cell line [13]. Here, we show that silencing of MCPIP1 expression partially protects HeLa cells against MG-132 toxicity, as revealed by MTT test, ATP content assay and propidium iodide staining. Thus, MCPIP1 expression contributes to the toxic properties of MG-132 toward HeLa cells.

Importantly, in HeLa cells the inhibition of NF-κB was shown to limit the proliferation and survival [43]. As shown here and in other studies, MCPIP1 inhibits NF-κB following treatment of cells with LPS, IL-1β [1, 9]. Noteworthy, besides cytokine- or LPS-stimulated NF-κB, MCPIP1 is also able to inhibit the constitutive activity of endogenous NF-κB. Vector-based studies on NF-κB-driven transcription clearly show that not only activated, but also basal NF-κB-dependent expression is inhibited by the overexpression of MCPIP1 (Fig. 4 in this manuscript, see also Fig. 6 in [1], Fig. 5b in [44] and Fig. 3b in [35]). In our study, MG-132 treatment decreased the activity of NF-κB, and the inhibition of MCPIP1 expression by siRNA restored NF-κB activity in MG-132-treated cells. Therefore, MCPIP1 is an inhibitor of NF-κB transcription factor, induced in MG-132-treated cells, which results in the inhibition of constitutive NF-κB. This action of MCPIP1 contributes to the toxicity of MG-132 in HeLa cells, which survival depends on increased constitutive NF-κB activity.

Due to its known NF-κB inhibitory and immunosuppressive properties, MCPIP1 represents a promising target in anti-cancer therapy. In this study, we provided initial and preliminary results, suggesting the possible toxic properties of MCPIP1 toward cancer cells and engagement of MCPIP1 in the toxicity of MG-132 proteasome inhibitor. We propose the interplay between MCPIP1 and NF-κB and its importance in regulation of cancer cell survival. However, more work needs to be done to verify the usefulness of this protein in therapeutical approaches.

## Materials and methods

### Materials

MG-132 inhibitor was from Tocris Bioscience. Epoxomicin was from Sigma Aldrich. Plastic materials were from BD Falcon. Both MG-132 and epoxomicin were dissolved in DMSO, thus as a control DMSO was used.

The genetic constructs encoding human MCPIP1 protein (cloned into pcDNA3.1*myc*/His (MCPIP1-*myc*) and MCPIP1 deprived of PIN/DUB domain cloned into pcDNA3.1*myc*/His vector (ΔPIN/DUB-*myc*)) were kindly provided by Danuta Mizgalska, PhD, Faculty of Biophysics, Biochemistry, and Biotechnology, Jagiellonian University, Krakow, Poland [11].

pNFκB-SEAP plasmid (Clontech), used for the studies on NF-κB-dependent gene expression, was a kind gift from Prof. Alicja Jozkowicz from the Faculty of Biophysics, Biochemistry, and Biotechnology, Jagiellonian University, Krakow, Poland.

#### Cell culture and treatment

Human hepatocellular liver carcinoma cell line (HepG2) and Human epithelial carcinoma cell line (HeLa) were purchased from ATCC collection. HepG2 cells from passages 95–105 were used. Both cell lines were cultured and seeded for the experiments as described before [13].

HepG2 cells stably transduced with a retroviral vector encoding GFP alone (HepG2-mock cell line), or encoding both GFP and dominant negative mutant of IκBα inhibitor (HepG2-IκBα(S/A) cell line), were kindly provided by Prof. Stephan Ludwig from Heinrich-Heine University, Düsseldorf, Germany [34]. The transduced cells were cultured in DMEM containing 1 g/l glucose and 5 % FBS in a humidified atmosphere at 37 °C and 5 % CO_2_ and passaged at c.a. 70–80 % of confluence.

#### Cell transfection

For MCPIP1-*myc* and ΔPIN/DUB-*myc* over-expression, cells were transfected with Lipofectamine 2000 (Life Technologies) according to manufacturer’s instructions. To reduce, the toxicity of Lipofectamine 2000 the medium was exchanged 4 h following transfection.

For MCPIP1 silencing new technology of modified and thus more stable Stealth RNAi siRNAs was used (Life Technologies). The cells were transfected with MCPIP1 specific siRNA (UAUCUUUGGUUUGUGGGUUUCCUUC, GC content 40 %) or low GC content negative siRNA (GC content 36 %) using Lipofectamine 2000 according to manufacturer’s recommendations. HeLa cells growing on Ø 60 mm cell culture dishes at the confluence of 40–50 % were transfected. For the transfection, 200 pmol of siRNA was mixed with 500 µl of Opti-MEM medium, and separately 10 µl of Lipofectamine 2000 was mixed with 500 µl of Opti-MEM and incubated for 5 min at room temperature. After that, Lipofectamine-containing Opti-MEM was added to siRNA-containing Opti-MEM and incubated at room temperature for 20 min. Then, the prepared mixture was added dropwise to the culture dish, the dish was rocked gently and incubated in cell culture incubator for 5 h. The medium was exchanged to fresh culture medium, and cells were cultured for additional 3 days and seeded for downstream experiments.

#### Western blotting

For the detection of proteins, total cell lysates were prepared using RIPA buffer (25 mM Tris•HCl, pH 7.6, 150 mM NaCl, 1 % NP-40, 1 % sodium deoxycholate, 0.1 % SDS) with protease inhibitor cocktail (Sigma Aldrich). For the analysis of phosphorylated proteins, RIPA buffer was additionally supplemented with phosphatase inhibitor cocktail (PhosSTOP, Roche). Protein separation on 12 % SDS-Page polyacrylamide gel and western blot procedure was performed as described before [13].

The following antibodies and dilutions were used: rabbit monoclonal anti-P-IκBα (Ser32) (1:2000, Cell Signaling, cat. 2859), rabbit monoclonal anti-IκBα (1:10 000, Abcam, cat. ab32518), rabbit polyclonal anti-MCPIP1 (1:3 000, GeneTex, cat. GTX110807), rabbit monoclonal anti-phospho-p65 (Ser536) (1:1,000, Cell Signaling, cat. 3033), rabbit polyclonal anti-p65 (0.5 µg/ml, Abcam, cat. ab16502), rabbit polyclonal anti-Myc-Tag (1:2 000, Cell Signaling, cat. 2272), mouse anti-α-tubulin (1:2 000, Calbiochem, cat. CP06), goat peroxidase-conjugated anti-rabbit (1:3 000, Cell Signaling, cat. 7074), and goat peroxidase-conjugated anti-mouse (1:20 000, BD Pharminogen, cat. 554002). The densitometry analysis was performed with ImageJ 1.40 g software [45].

#### Secreted alkaline phosphatase (SEAP) activity assay

For the analysis of NF-κB-driven gene expression, a secreted alkaline phosphatase (SEAP)-based assay was performed. HepG2 or HeLa cells were seeded on 48-well plates and transfected with vectors encoding MCPIP1-*myc* or ΔPIN/DUB-*myc* protein or empty vector (0.1 µg (30 % of total DNA mass) or 0.05 µg (15 %) of total DNA mass) together with reporter pNFκB-SEAP plasmid (0.15 µg) and the empty vector (0.05 or 1 µg, depending on the amount of MCPIP1-encoding vector) to give final DNA amount: 0.3 µg. Two days following transfection, the cells were stimulated with IL-1β in the total medium volume of 300 µl for additional 24 h and the enzymatic activity of SEAP in the medium was measured using Phospha-Light System (Applied Biosystems). The assay was performed following the manufacturer’s instructions, but reagents volumes were limited to 40 %. During the procedure, the medium was diluted three times with 1 × *Dillution Buffer*. The results were normalized to the survival of transfected cells measured by MTT assay (the survival was: for HepG2 cells, 15 % MCPIP1-*myc* plasmid: 102.3 ± 11.4 %, 30 % MCPIP1-*myc* plasmid: 94.4 ± 1.4 %; for HeLa cells, 15 % MCPIP1-*myc* plasmid: 97.0 ± 2.8 %, 30 % MCPIP-*myc* plasmid: 94.2 ± 5.8 %, 15 % ΔPIN/DUB-*myc* plasmid: 104.2 ± 7.5).

#### Cell cytotoxicity and apoptosis assays

For MTT cell viability assay, siRNA-transfected HeLa cells (see above) were seeded on 96-well transparent plates. The cells were treated with MG-132 for 24 h. Thiazolyl Blue Tetrazolium Bromide (MTT, Sigma) was added for additional 60 min in a final concentration of 500 ng/ml. The medium was removed by suction and MTT crystals were dissolved in acidic (40 mM HCl) isopropanol. The absorbance was measured with Infinite 200 microplate reader (Tecan Group Ltd.) at 570 nm with the reference wavelength 650 nm.

ATP content measurement was performed using *ATPLite* kit (PerkinElmer). siRNA-transfected HeLa cells were seeded on 96-well white plates and treated with MG-132 for 24 h.. Then, 50 µl of *mammalian cell lysis solution* was added to each well containing cells in 100 µl of culture medium, and the plate was shaken for 5 min in an orbital shaker at 300 rpm to lyse the cells. Following cell lysis, 50 µl of resuspended, room temperature-adjusted *substrate solution* was added to each well and plate was shaken again for 5 min at 300 rpm. After that, the plate was dark-adapted and luminescence was measured using *SPECTRAFluor Plus* (Tecan) microplate reader.

The percentage of dead cells was measured using double staining with propidium iodide (PI) and Hoechst 33342. For this, siRNA-transfected cells were seeded on 24-wells plates and treated with MG-132 for 24 h. Then, 5 µg/ml of Hoechst 33342 was added to the culture media for 30 min, and 1 µg/ml of PI for the last 5 min. The cells were imaged using microscope and *Nikon D5000* camera. To avoid any bias, the process of total cells (Hoechst staining) and dead cells (PI staining) counting was automated using *ImageJ* software [45]. Using this method, a mean nuclei counts per picture (Hoechst staining) was 908 for DMSO-treated cells and 443 for MG-132-treated cells, and mean dead cells count per picture (PI staining) was 12 for DMSO-treated cells and 59 for MG-132 treated cells. For each data point showed in the graph, nine photographs were analyzed (three biological replicates performed in three technical replicates; for statistics *n* = 3).

Caspase 3/7 activity was measured using Caspase-Glo 3/7 Assay (Promega). siRNA-transfected cells were seeded on white 96-wells plates and treated with MG-132 for 24 h. Next, 50 µl of Caspase-Glo 3/7 Reagent was added directly to the wells containing 50 µl medium. The plates were shaken for 1 min at 300 rpm and kept in the dark at room temperature for 90 min. Luminescence was measured with *Infinite M200* microplate reader.

## Abbreviations

MCPIP1: Monocyte chemotactic protein-1 induced protein 1
NF-κB: Nuclear factor-κB
IL: Interleukin
IκB: Inhibitor of NF-κB
